# Intraglandular Extirpation of Goitre of Socin’s Method

**Published:** 1888-12

**Authors:** 


Intraglandular Extirpation of Goitre of Socin's Method promises to become the leading method of the future for all cases where there is neither a general tumefaction of the thyroid gland (diffuse hyperplasis), nor a multilocular development of goitreous nuclei. In these two latter cases the iodine injections will retain their value. This is the conclusion of Edmond Vignard in *Le Progrès Médical* of October 13, 1888. With good reason he fears that Woelfler's method of tying the four thyroid arteries will lead to cachexia strumipriva, besides of the difficulty of its execution, whereas nothing is simpler than intraglandular extirpation. Incision through the thyroid tissue till you reach the bluish sheath of the goitre, shelling out and enucleation of the tumor with the fingers and scalpel-handle. The number of arterial twigs to be tied is mostly small. If there are several new formations, each can be treated the same way. Then follows drainage and stitching. Among twenty-nine operations Socin could follow this plan successfully twenty-six times. Palpation reveals whether there are separate tumors within the gland or general hyperplasis. Vignard does not mention the injections of Fowler's solution.

